# Natalizumab-associated Progressive Multifocal Leukoencephalopathy in Patients with Multiple Sclerosis: A Mini Review

**DOI:** 10.7759/cureus.3093

**Published:** 2018-08-03

**Authors:** Muniba Fayyaz, Syeda S Jaffery

**Affiliations:** 1 Internal Medicine, Fatima Memorial Hospital, Lahore, PAK; 2 Research, CiBNP, Fairfield, USA

**Keywords:** progressive multifocal leukoencephalopathy, natalizumab, disease modifying therapy

## Abstract

Progressive multifocal leukoencephalopathy is a fatal demyelinating disease caused by the John Cunningham virus. It causes white matter inflammation in multiple areas of the brain. Although rare, it has been known to occur in patients with immunodeficiency states and those taking chronic immunosuppressive medications for diseases such as multiple sclerosis and psoriasis. In this article, we will discuss about the drug-induced progressive multifocal encephalopathy and its diagnosis and management in patients taking natalizumab and other disease-modifying therapies among patients with multiple sclerosis.

## Introduction and background

Progressive multifocal leukoencephalopathy (PML) is a rare life-threatening disease, caused by the reactivation of John Cunningham (JC) virus. The JC virus is a small ubiquitous DNA type of polyomavirus with a circular double-stranded DNA [1]. It is characterized by progressive damage or inflammation of the white matter of the brain in multiple areas causing lytic infection of glial cells in severely immunosuppressed patients. It occurs in 0.2 cases per 100,000 persons in the general population. After asymptomatic primary infection which occurs in childhood, the virus remains quiescent in the kidneys, bone marrow, and lymphoid tissue. It reactivates as the patient’s immune system is suppressed most likely seen in patients with human immunodeficiency virus (HIV) (80%), lymphoid malignancies (13%) and those taking immunosuppressants (5%) [2-5]. There are different treatment options for PML according to immune status and other medical conditions of the patient [6]. Drug-associated PML is the most common these days especially in patients taking immunomodulating drugs. The most common drug involved is natalizumab. Natalizumab is a highly efficacious treatment for relapsing forms of multiple sclerosis (MS). Patients who are seropositive for the anti-JC virus antibody and exposed to multiple doses of natalizumab therapy for more than two years, are at a higher risk for developing PML [7]. In this article, we will discuss about the drug-induced PML especially as an adverse effect of natalizumab.

## Review

Progressive multifocal leukoencephalopathy has been an adverse reaction to many drugs in the past. Some of the drug classes that have been identified to cause this demyelinating disease include immunosuppressants, antineoplastics, and corticosteroids. It is important for the prescribers and patients to be aware of this fatal central nervous disease’s adverse effect and it is also recommended that patients undergo regular testing to monitor for its presence [8-9]. Many studies have supported the presence of PML as an adverse effect to these classes of drugs. Maas et al. conducted a study collecting 326 cases of drug-associated PML and compared it between MS, neoplasm, post-transplantation, and autoimmune cases. This study concluded that the rate of drug-induced PML occurred mainly in MS and neoplasms after an average of 29.5 months. The main symptoms included motor weakness and cognitive impairment; however, dysarthria and ataxia were also reported. The lesions occurred mostly in the supratentorial region within the frontal and parietal lobes. The JC virus tested positive in the first cerebrospinal fluid (CSF) sample in 63.5% of cases and conversion after one or more negative occurred in 13.7% of cases. Some 52.2% of patients died, ranging from 12.0% to 83.3% in the MS and neoplasm subgroups, respectively [10]. Oshima et al. identified that the risk PML with natalizumab was higher than any other disease-modifying drugs used in MS. Other medications that caused PML in MS include fingolimod, mycophenolate mofetil, rituximab, alemtuzumab, and dimethyl fumarate. No association was reported with glatiramer acetate, interferon, and dalfampridine [11-12]. Another study supporting these findings was carried out by Chalkley and Berger which demonstrated that the risk of PML was high with natalizumab and efalizumab. While efalizumab (lymphocyte function-associated antigen 1 inhibitor) was used for psoriasis, it has now been withdrawn from the market. Meanwhile, natalizumab (α4β1 and α4β7 integrin inhibitor) is widely used in the treatment of MS. From 2005 to 2013, there have been a total of 400 cases of PML associated with natalizumab. PML associated with the use of therapeutic agents, especially, natalizumab, does share similarities with HIV-related PML; however, distinct differences exist. Radiographically isolated PML is seen more commonly with natalizumab-associated PML and the disease appears to be heralded more often by cognitive and behavioral disturbances. Furthermore, the mortality of natalizumab-associated PML is substantially lower [12]. In addition, Maillart et al. demonstrated the clinico-radiologic outcomes in patients with MS taking disease-modifying therapies (DMTs) after suffering from natalizumab-associated PML. They concluded that no clinical worsening and relapse of PML symptoms were reported with fingolimod and dimethyl fumarate. Also, there was no radiologic worsening of natalizumab-associated PML at the end of the follow up [13]. The risk of drug-induced PML is increased especially if a patient has been on immunosuppressants in the past; however, its occurrence without previous therapy with DMT is still possible. When treating a patient with natalizumab it is important that clinicians stratify the risk by employing JC-virus antibody status, prior immunosuppression, and length of natalizumab treatment. These tools can help minimize the risk of developing PML.

The diagnosis and management of PML

It remains a challenge for clinicians to identify the patients with natalizumab-associated PML; however, unique clinical features, sensitive laboratory analyses, and advanced magnetic resonance imaging (MRI) which shows contrast enhancement can aid in its identification. A laboratory test that could aid in the diagnosis of PML can include C-reactive protein (CRP). Lanzillo et al. demonstrated the use of CRP in natalizumab-treated MS patients who underwent the JC virus stratify antibody test to measure the serum ultrasensitive C-reactive protein (usCRP) levels, and to perform blood and urine JC virus PCR. They concluded that the level of usCRP was higher in urinary JCV DNA-positive patients and correlated with the number of DNA copies in urine. Also, JC viruria was significantly correlated with a high JC virus antibody index and high serum usCRP levels suggesting that usCRP may be a useful marker of JC virus reactivation [14]. Fortunately, natalizumab-associated PML remains a rare entity compared to MS-associated disabilities, and the risk may be mitigated with an appropriate patient selection, accurate and rapid diagnosis, and aggressive treatment strategies. Also, the diagnosis of drug-induced PML is done by the detection of JC virus in CSF. The management of PML has routinely used plasma exchange (PLEX) or immunoabsorption for the rapid clearance of natalizumab and it shortens the action period of natalizumab (usually several months). Exacerbation of symptoms and enlargement of lesions on MRI have occurred within a few days to a few weeks after PLEX, indicative of immune reconstitution inflammatory syndrome (IRIS). IRIS is an exuberant inflammatory response that increases the damage caused by JC virus infection and ultimately leads to a combined PML/IRIS syndrome. This syndrome seems to be more common and more severe in patients with natalizumab-associated PML than it is in patients with HIV-associated PML. It is important that we manage the IRIS reaction in order to improve the outcomes. The current management of IRIS also includes use of PLEX for the elimination of natalizumab and reconstitution of immune surveillance, although this may lead to a rebound MS activity and an increase in IRIS. Furthermore, in a study done by Hodecker et al. it was demonstrated in two patients with PML/IRIS that the use of maraviroc instead of PLEX improved IRIS. Moreover, it is crucial to monitor the risk incurred during the use of natalizumab beyond three years. Overall, the current prophylaxis and treatment of PML can include immune reconstitution, restoration of the immune response of the JC virus, and suppression of IRIS. Even though, this management approach reduced the incidence and improved the survival of patients with PML (especially HIV patients), the outcome in most of the PML patients is still relatively poor [15-18]. 

## Conclusions

Progressive multifocal leukoencephalopathy is a fatal demyelinating disease that can present as an adverse effect of DMTs used in MS especially natalizumab. The prognosis is poor regardless of the immune status of the patient. Although it is a rare entity, its occurrence, diagnosis, and management should be explained to a patient taking these drugs. Overall, more research is required to support and validate our article.
